# Modeling antiviral response in the liver using human pluripotent stem cell-derived macrophages

**DOI:** 10.1093/lifemedi/lnae001

**Published:** 2024-01-12

**Authors:** Yaxuan Zhang, Xingwu Zhang, Fuyu Duan, Huimin Qiao, Mingli Gong, Hui Qiu, Xia Chen, Peiliang Wang, Yuan He, Qiang Ding, Jie Na

**Affiliations:** Center for Stem Cell Biology and Regenerative Medicine, School of Medicine, Tsinghua University, Beijing 100084, China; Center for Stem Cell Biology and Regenerative Medicine, School of Medicine, Tsinghua University, Beijing 100084, China; Center for Stem Cell Biology and Regenerative Medicine, School of Medicine, Tsinghua University, Beijing 100084, China; Cord Blood Bank, Guangzhou Institute of Eugenics and Perinatology, Guangzhou Women and Children’s Medical Center, Guangzhou Medical University, Guangzhou 510000, China; Center for Infectious Disease Research, School of Medicine, Tsinghua University, Beijing 100084, China; Center for Infectious Disease Research, School of Medicine, Tsinghua University, Beijing 100084, China; Center for Stem Cell Biology and Regenerative Medicine, School of Medicine, Tsinghua University, Beijing 100084, China; Center for Stem Cell Biology and Regenerative Medicine, School of Medicine, Tsinghua University, Beijing 100084, China; Center for Stem Cell Biology and Regenerative Medicine, School of Medicine, Tsinghua University, Beijing 100084, China; Boehringer Ingelheim-Tsinghua University Joint Research Center for Immuno-Infection, Tsinghua University, Beijing 100084, China; Center for Infectious Disease Research, School of Medicine, Tsinghua University, Beijing 100084, China; Center for Stem Cell Biology and Regenerative Medicine, School of Medicine, Tsinghua University, Beijing 100084, China

Dear Editor,

Kupffer cells (KCs) are tissue-resident macrophages (TRMs) in the liver sinusoids. KCs comprise the largest TRM populations in the body and play essential roles in the innate immune response [1]. To model antiviral response in the liver, we first obtained CD45^+^CD14^+^CD163^+^ macrophages (iMACs) from human pluripotent stem cells (hPSCs) through CD34^+^CD43^+^ hematopoietic stem/progenitor cell (HSPC) stage (Fig. 1A–C) [2]. To resolve the heterogeneity of iMAC differentiation, we performed single-cell RNA sequencing (scRNA-seq) and single-cell assay for transposase-accessible chromatin sequencing (scATAC-seq) of Day 14 differentiation cultures, which consisted primarily of monocytes and macrophages. Cells fell into two large groups in scRNA-seq and scATAC-seq analysis (Fig. 1D and 1E). Granulocyte–macrophage progenitor (GMP) (7.6%), proliferating GMP (pro-GMP) (7.2%), monocyte (Mono) (5.7%), proliferating Mono (pro-Mono) (6.6%), monocyte transiting to macrophage (Mono-Mac) (8.7%), macrophage (Mac) (13.6%), and proliferating Mac (pro-Mac) (10.7% and 8%) cell types comprise the majority (about 70%) of the Day 14 differentiation cultures. In contrast, the smaller cell group consisted of eosinophils (Eos), erythrocyte (Ery), and megakaryocyte (Mk) clusters (Fig. 1D–F). We also examined marker gene expressions in each cell cluster. Macrophage lineage genes *CSF1R*, *MAF*, and *MRC1* were highly expressed in Mono-Mac, Mac, pro-Mac 1 and 2 clusters (Figs. 1G and S1A). Accordingly, their chromatin was more accessible in these cell clusters according to scATAC-seq (Fig. 1H). We took out granulocyte–macrophage lineage cell clusters for closer examination. From the UMAP plot, pro-GMP, GMP, GMP-Mono, pro-Mono, Mono, Mono-Mac, pro-Mac, and Mac clusters formed a continuum (Fig. 1I). RNA velocity analysis also showed one differentiation path from pro-GMP, pro-Mono to pro-Mac 1 and 2, and another from GMP, GMP-Mono, Mono, Mono-Mac to Mac (Fig. 1J). This result could represent a fraction of fast proliferating cells and the rest of less proliferative cells within each cell type. Cell cycle analysis showed that about 50% of pro-GMP and pro-Mac 1 were in the G2/M phase, and the other 50% were in the S phase, indicating they were actively dividing (Fig. S1B). Conversely, no G2/M-phase cells existed in GMP-Mono, Mono, Mon-Mac, and Mac clusters (Fig. S1B).

**Figure 1.**
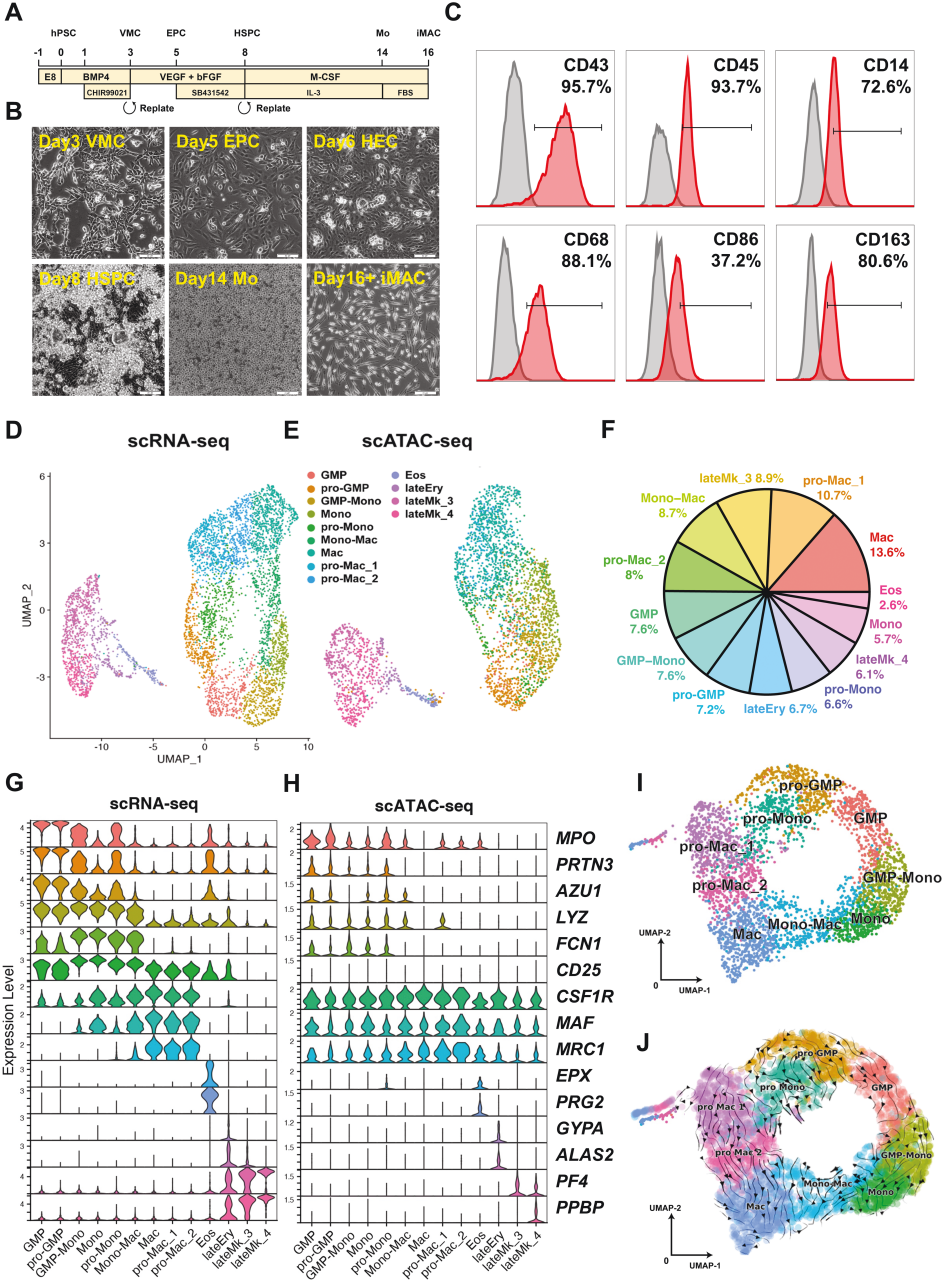
**scRNA-seq and scATAC-seq analysis of hPSC-derived macrophages.**(A) Schematics of the differentiation protocol. (B) Images of cells at different differentiation stages. Scale bar, 20 μm. (C) Flow cytometry (FCM) analysis of macrophage surface marker expression. Tests were performed more than three times as biological replicates, and the figures show the representative one. (D) UMAP of scRNA-seq. (E) UMAP of scATAC-seq. (F) Percentage of different cell types based on scRNA-seq analysis. (G) Violin plot of marker gene expression in different cell clusters based on scRNA-seq. (H) Violin plot of marker genes’ open chromatin peak enrichment in different cell clusters based on scATAC-seq. (I) Cell cluster analysis of macrophage-related populations. (J) Cell fate trajectory analysis of macrophage-related population using RNA velocity.

Tissue environments play pivotal roles in shaping the transcriptome and epigenome landscapes of TRMs including KCs [3, 4]. We set up a hepatic cell and macrophage co-culture assay (Fig. 2A and 2B). Huh7 cells (hepatocellular carcinoma cells) or primary human hepatocytes (pHHs) were first seeded. Twelve hours later, iMACs were added and co-cultured for 72 h. Immunofluorescence showed that iMACs highly expressed macrophage-specific marker CD163 (Fig. 2A and 2B). In pHH co-culture, iMACs also had elongated morphology and expressed CD163 and CD68 (Fig. 2B). Transcriptome analysis confirmed the identities of iMACs, pHHs, and Huh7 cells (Fig. S2). Gene ontology (GO) analysis and gene set enrichment analysis (GSEA) revealed iMACs up-regulated gene expressions related to wound healing, liver development, metabolic process, etc., after co-cultured with hepatic cells and became similar to KCs [5, 6] (Fig. S3).

**Figure 2.**
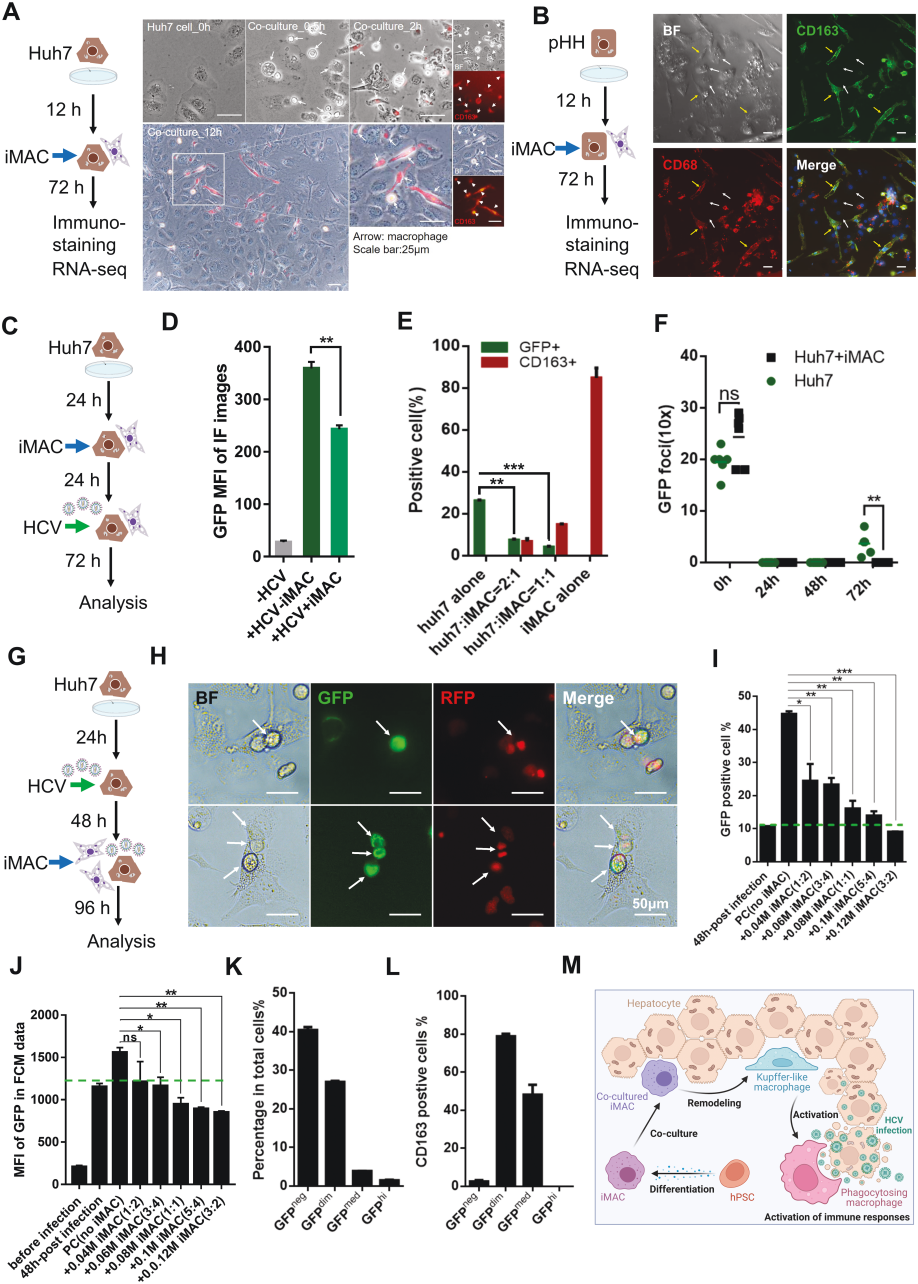
**iMAC inhibition of HCV infection in hepatic cells.**(A) Schematic illustration of the Huh7-iMAC co-culture pipeline with brightfield microscopy and immunostaining of Huh7-iMAC co-culture. Scale bar, 25 μm. Arrows indicate iMACs in the Huh7-iMAC co-culture. (B) Schematic illustration of the pHH-iMAC co-culture pipeline with brightfield microscopy and immunostaining of pHH-iMAC co-culture. Scale bar, 50 μm. White arrows indicate the pHHs and yellow arrows indicate the iMACs in the pHH-iMAC co-culture. (C) Schematics of HCV infection after the Huh7 cells were co-cultured with iMACs. (D) GFP MFI in total cells after 3-day GFP-HCV infection of Huh7 cells with or without iMACs. (E) Proportions of the GFP-positive infected Huh7 cells and the CD163-positive iMACs in different co-culture conditions. (F) GFP foci number in the infection supernatants at different time points in the co-cultured or mono-cultured Huh7 cells. (G) Schematics of HCV infection 2 days before the Huh7 cells were co-cultured with iMACs. (H) Brightfield microscopy and immunostaining of the infected Huh7 cells after they were co-cultured with iMACs. Arrows indicate the GFP-positive Huh7 cells infected by HCV and engulfed by iMACs. Scale bar, 50 μm. (I) Proportions of the GFP-positive infected Huh7 cell population in total RFP-positive Huh7 cells under different co-culture conditions. (J) GFP MFI of the GFP-positive Huh7 populations in (I) under different co-culture conditions. (K) Percentages of the GFP-positive cell populations in total cells of the Huh7-iMAC co-culture. (L) Percentages of the CD163-positive cell populations in total cells with different GFP intensities. (M) Graphical summary of the study. Created with BioRender.com. *N* = 3 biological replicates for (D–F) and (I–L) and the column or scatter plots show the mean values of the results, and the bars represent the standard deviation of each group. Independent *t*-test was used for significance analysis in (D–F). One-way ANOVA and multiple *t*-test were used for significance analysis in (I–J). * *P* < 0.05, ** *P* < 0.01, *** *P* < 0.001, and “ns” represents “no significance.” All statistical analyses were performed by Prism 7.

We next investigated whether iMACs could inhibit hepatitis C virus (HCV) infection of hepatic cells. Huh7 cells highly susceptible to HCV infection were modified to express H2B-RFP to be distinguished from iMACs. Recombinant HCV expressing green fluorescent protein (GFP) was also engineered. Thus, HCV-infected Huh7 cells would have nuclei labeled in red and cytoplasm in green fluorescence. First, we tested whether iMACs could inhibit HCV infection of Huh7 cells. iMACs were added at different ratios to Huh7 cells 24 h after seeding. After 24 h co-culture, GFP-HCV was added, and cells were cultured for another 72 h and subjected to several analyses (Fig. 2C). Remarkably, iMACs significantly reduced the GFP mean fluorescence intensity (MFI) in Huh7 cells (Fig. 2D). With an increased ratio of iMACs to Huh7 cells, the proportion of CD163^+^ cells rose, and HCV-infected GFP^+^ Huh7 cells dropped (Fig. 2E). Such inverse correlation strongly suggested that the iMACs inhibited HCV infection in a cell number-dependent manner. Moreover, iMACs could also inhibit infected Huh7 cells from releasing HCV, as significantly fewer GFP foci were observed within iMAC-Huh7 co-culture supernatant (Fig. 2F, 72 h). These results demonstrated that iMACs in the Huh7 culture could robustly inhibit HCV infection and release.

Next, we investigated whether iMACs could engulf the infected Huh7 cells, e.g. cure the infection. Huh7 cells were infected with GFP-HCV for 48 h. Afterward, the infected Huh7 cells were washed extensively and re-seeded alone or with iMACs at different ratios. These cells were grown for 96 h before FCM analysis (Fig. 2G). We observed numerous cells with red nuclei and green cytoplasm inside iMACs (Fig. 2H), suggesting that iMACs could indeed engulf infected Huh7 cells. After the 48-h infection, about 10% of Huh7 cells were GFP-positive (Fig. 2I). Without iMACs, the proportion of GFP^+^ cells in the RFP^+^ Huh7 cells reached nearly 45% after 96 h. In the presence of iMACs, GFP^+^ Huh7 cells decreased significantly, and the GFP^+^ proportion in the RFP^+^ Huh7 cells was also negatively correlated with the ratio of iMAC/Huh7. When the ratio was 1.5, the percentage of GFP^+^ cells was lower than 10% (Fig. 2I). The GFP MFI in GFP^+^ cells also decreased in co-culture groups (Fig. 2J), indicating that the HCV load in co-cultured Huh7 cells might be milder than that in Huh7 cells alone. Total cells in the infected co-cultures could be divided into four subgroups based on their GFP intensity. Forty percent of cells are GFP^neg^, GFP^dim^ cells accounted for more than 25%, and the GFP^mid^ and GFP^hi^ cells comprised less than 10% of total cells (Fig. 2K). Interestingly, about 80% of GFP^dim^ cells and 50% of GFP^mid^ cells had macrophage-specific marker CD163 expressions (Fig. 2L), indicating that these cells were iMACs ingesting infected Huh7 cells. Therefore, iMACs could relieve or cure the HCV infection by engulfing and eliminating infected cells.

We also tested whether secreted factors from iMACs could protect Huh7 cells from HCV infection. Huh7 cells already infected 2 days with GFP-HCV were cultured alone, co-cultured with iMACs, or cultured in different chambers of transwell plate for 96 h (Fig. S4A). Only when Huh7 cells contacted directly with iMACs could a significant decrease in GFP^+^ cells be detected (Fig. S4B). Thus iMAC inhibition of infection was independent of secreted factors.

Gene expression analysis found that 189 and 698 genes were up- and down-regulated when iMACs were co-cultured with HCV-infected Huh7 cells compared with those co-cultured with non-infected Huh7 cells (Fig. S5A). Up-regulated genes were associated with immune responses such as immune cell recruitment, phagocytosis, and cytotoxicity (Fig. S5B). In contrast, 187 and 189 genes were up- and down-regulated when iMACs encountered HCV versus iMACs alone (Fig. S5C). Most up-regulated genes were associated with the regulation of virus-related processes (Fig. S5D). In summary, different pathways were enriched when iMACs encountered HCV with or without Huh7 cells, indicating different antiviral responses in iMACs were triggered by HCV-infected cells or HCV alone.

We also tested whether iMACs had a similar inhibitory effect on hepatitis B virus (HBV) in an iMAC-hepatic cell co-culture. In this case, we constructed HepG2 cells to express Na^+^ taurocholate cotransporting polypeptide (NTCP), an essential receptor for HBV recognition and entry of the hosts [7], and confirmed that HepG2-NTCP cells could be infected by HBV (Fig. S6A–E). Moreover, HBV infection in HepG2-NTCP cells could be inhibited by entecavir (ETV), a previously validated inhibitor of HBV (Fig. S6F–H). As for the iMAC effects on HBV infection, HepG2-NTCP cells were inoculated with HBV and then cultured for 3 days. Then iMACs were added into the culture at different ratios and co-cultured for 6 days (Fig. S6I). However, iMACs did not reduce the HBV core DNA level (Fig. S6J). The HBV surface antigen (HBsAg) level slightly decreased with the increase of iMAC/HepG2-NTCP ratio at 7 and 9 days post-infection (Fig. S6K), indicating that the inhibitory effect of iMACs towards HBV infection was mild. We speculated that iMACs in the infected co-culture might be able to clear some of the free HBV virions in the supernatant but could not inhibit the HBV replication in the HepG2-NTCP cells.

Figure 2M summarizes the findings of our study. Single-cell map of hPSC-derived iMACs validated that by Day 14, most cells in our differentiation culture were monocyte-macrophages. Upon co-culture with human hepatic cells, iMACs adapted to the tissue microenvironment, transformed into KC-like cells, and displayed corresponding gene expression changes. KC-like iMACs showed a strong inhibitory effect on HCV, which depended on iMACs in direct contact with infected cells. Our results suggested that hPSC-derived iMACs could be a useful model for studying the immunopathogenic response in liver infection.

## Research limitations

It is challenging to study HBV infection in our pHH-iMAC co-culture system as pHHs degenerated rapidly after 3 days. Although there are small molecule cocktails that could maintain pHHs for up to 1 month, their effects on KCs and iMACs are unknown. Moreover, HBV infection and replication take 72 h or longer, and the process of HBV infection seems more discrete than HCV infection. Thus iMACs did not show a significant inhibitory effect. Adding other immune cell types with iMACs may better protect cells against HBV infection.

## Supplementary Material

lnae001_suppl_Supplementary_Figures_S1

lnae001_suppl_Supplementary_Figures_S2

lnae001_suppl_Supplementary_Figures_S3

lnae001_suppl_Supplementary_Figures_S4

lnae001_suppl_Supplementary_Figures_S5

lnae001_suppl_Supplementary_Figures_S6

lnae001_suppl_Supplementary_Material
