# Supplementary figures and images for: Modeling antiviral response in the liver using human pluripotent stem cell-derived macrophages
Source: Life Med. 2024 Jan 12;3(1):lnae001. doi: 10.1093/lifemedi/lnae001 (PMC11749128; doi:10.1093/lifemedi/lnae001)

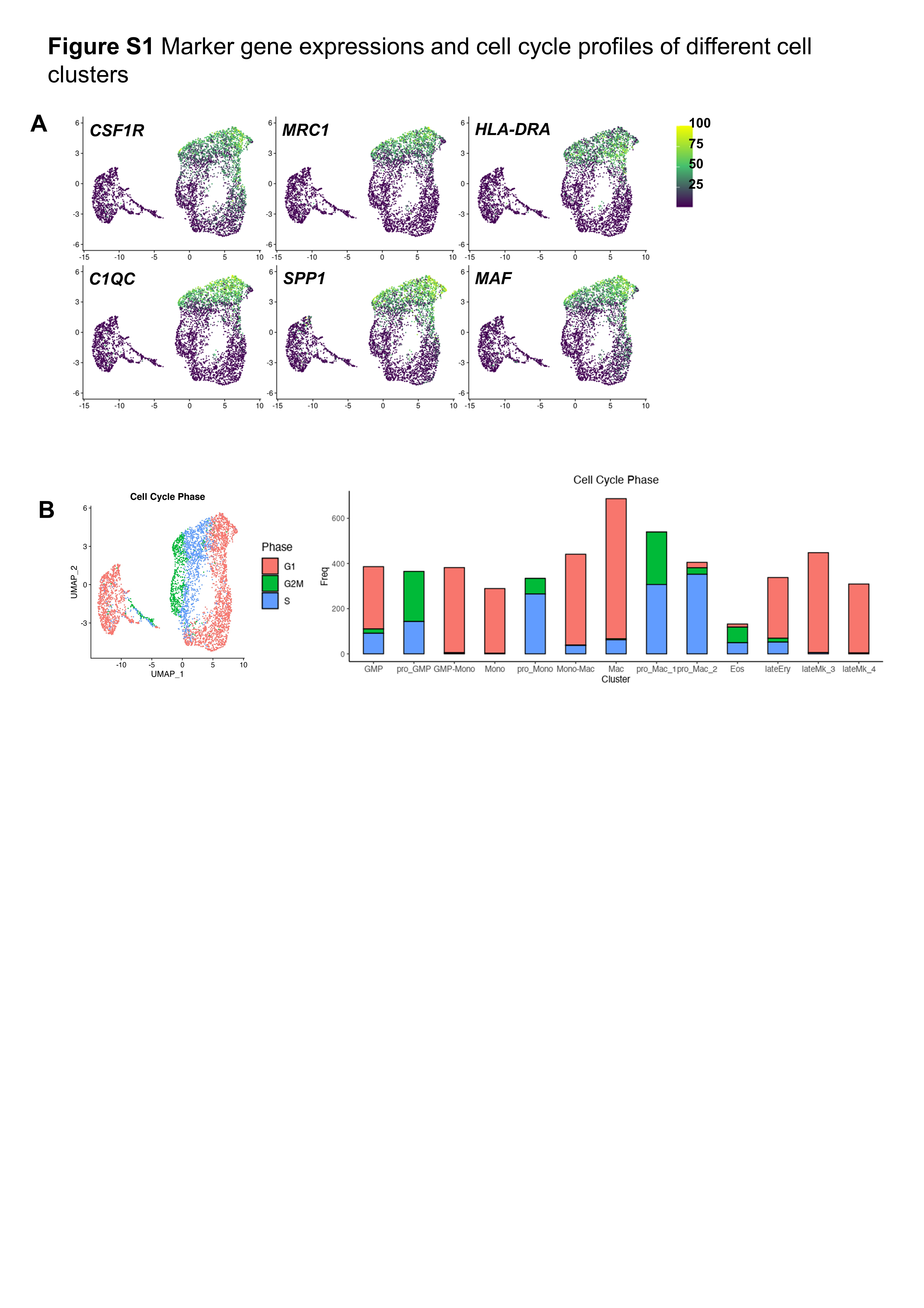

Supplement: lnae001_suppl_Supplementary_Figures_S1 [file lnae001_suppl_Supplementary_Figures_S1.TIF]

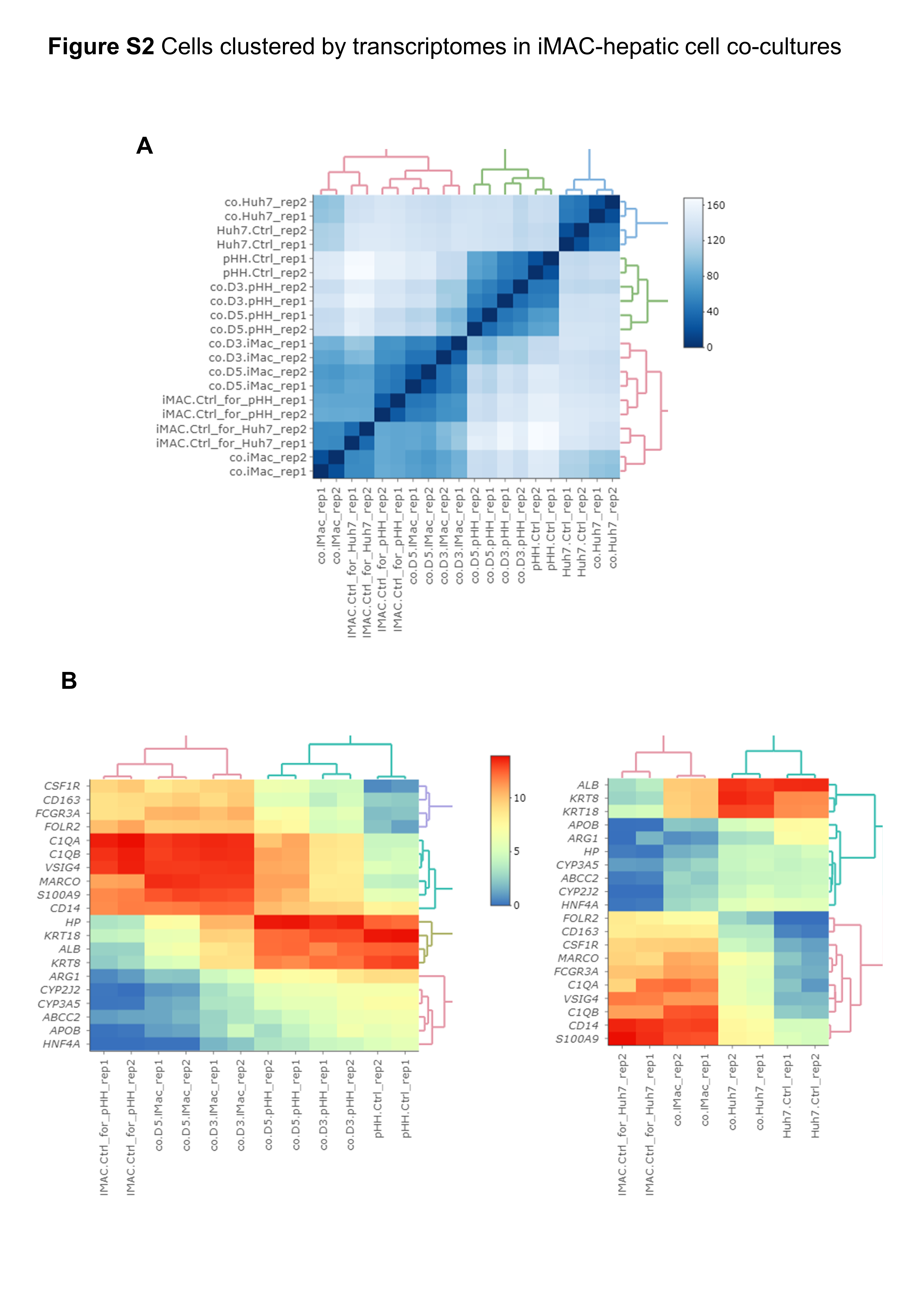

Supplement: lnae001_suppl_Supplementary_Figures_S2 [file lnae001_suppl_Supplementary_Figures_S2.TIF]

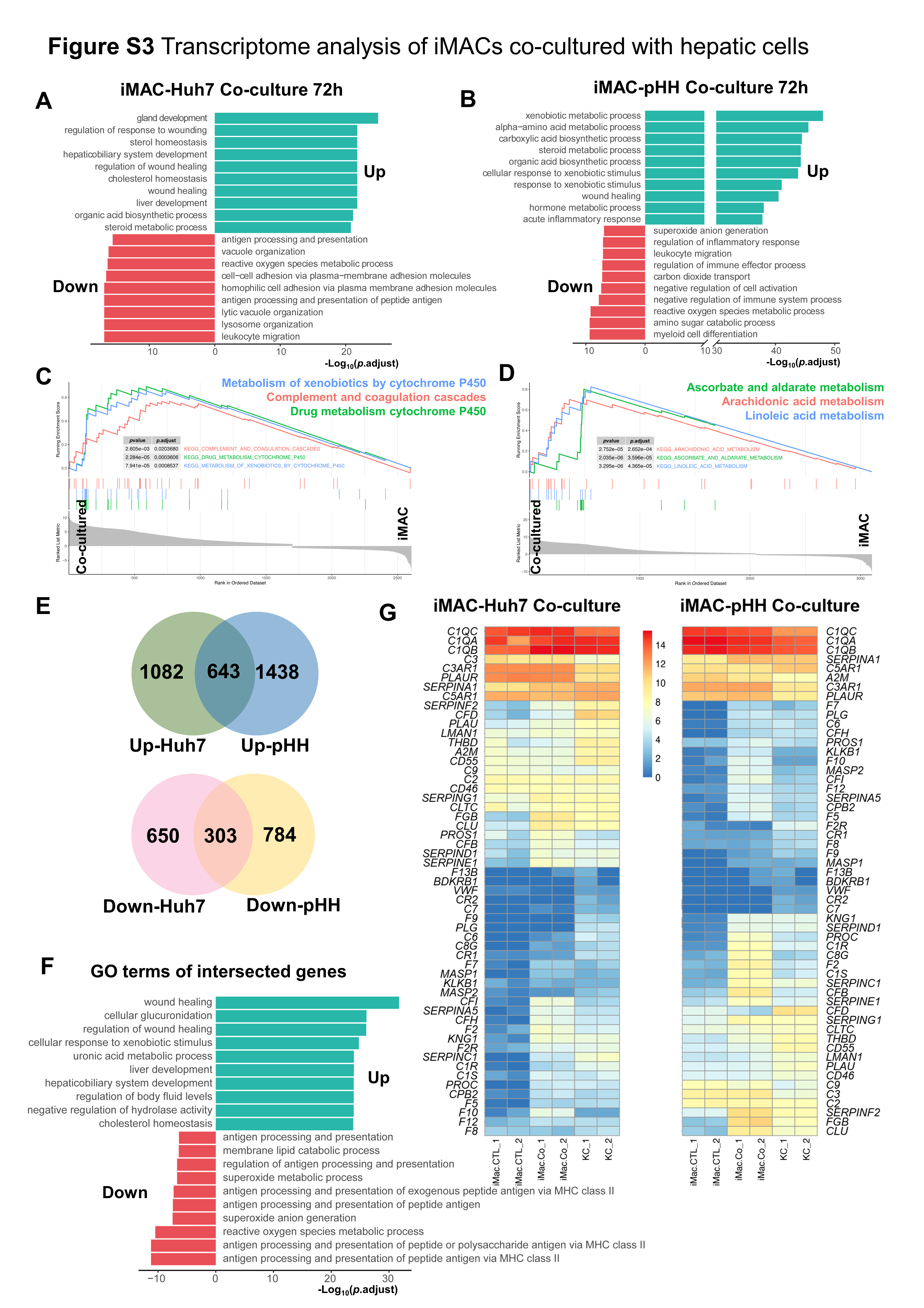

Supplement: lnae001_suppl_Supplementary_Figures_S3 [file lnae001_suppl_Supplementary_Figures_S3.TIF]

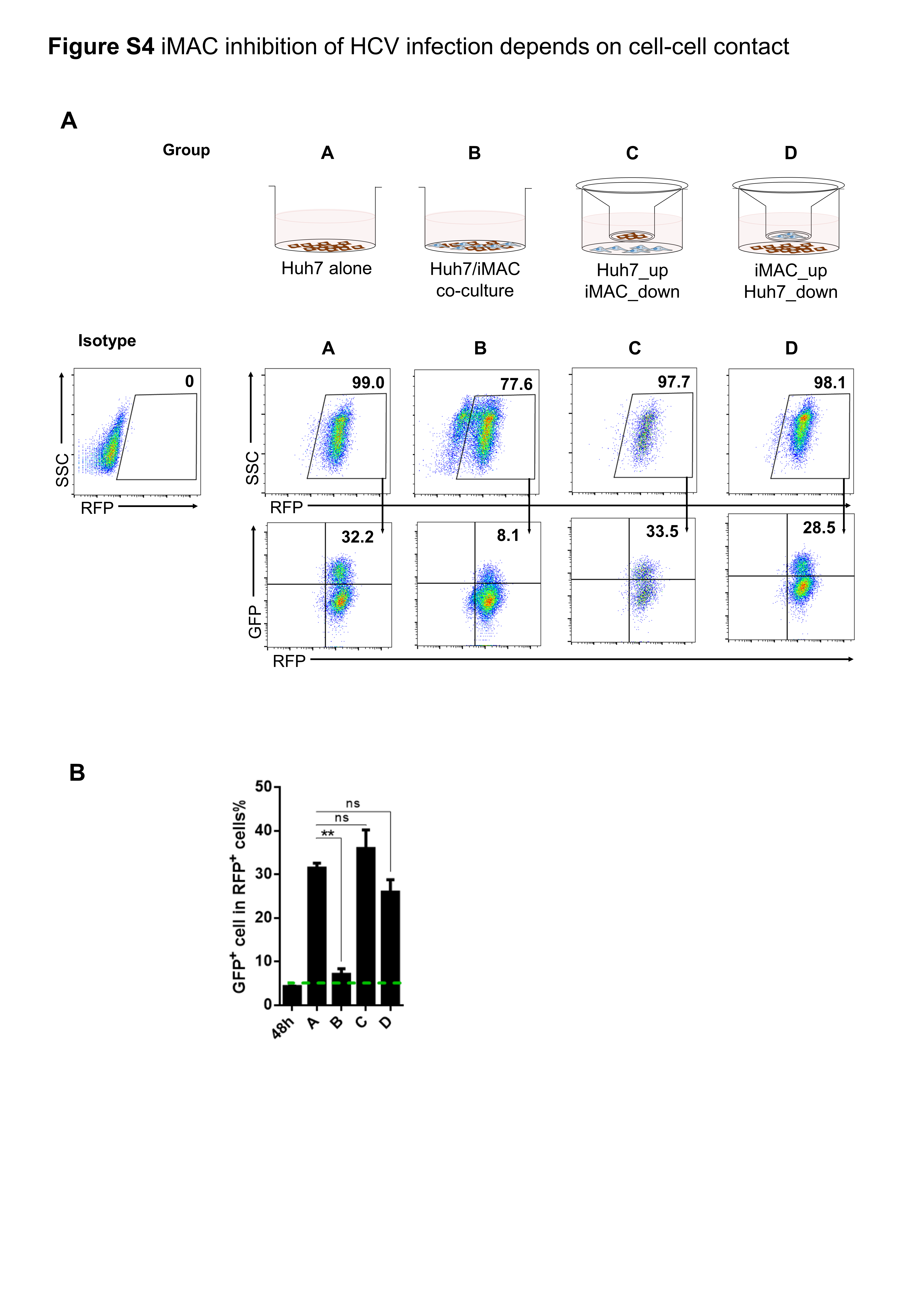

Supplement: lnae001_suppl_Supplementary_Figures_S4 [file lnae001_suppl_Supplementary_Figures_S4.TIF]

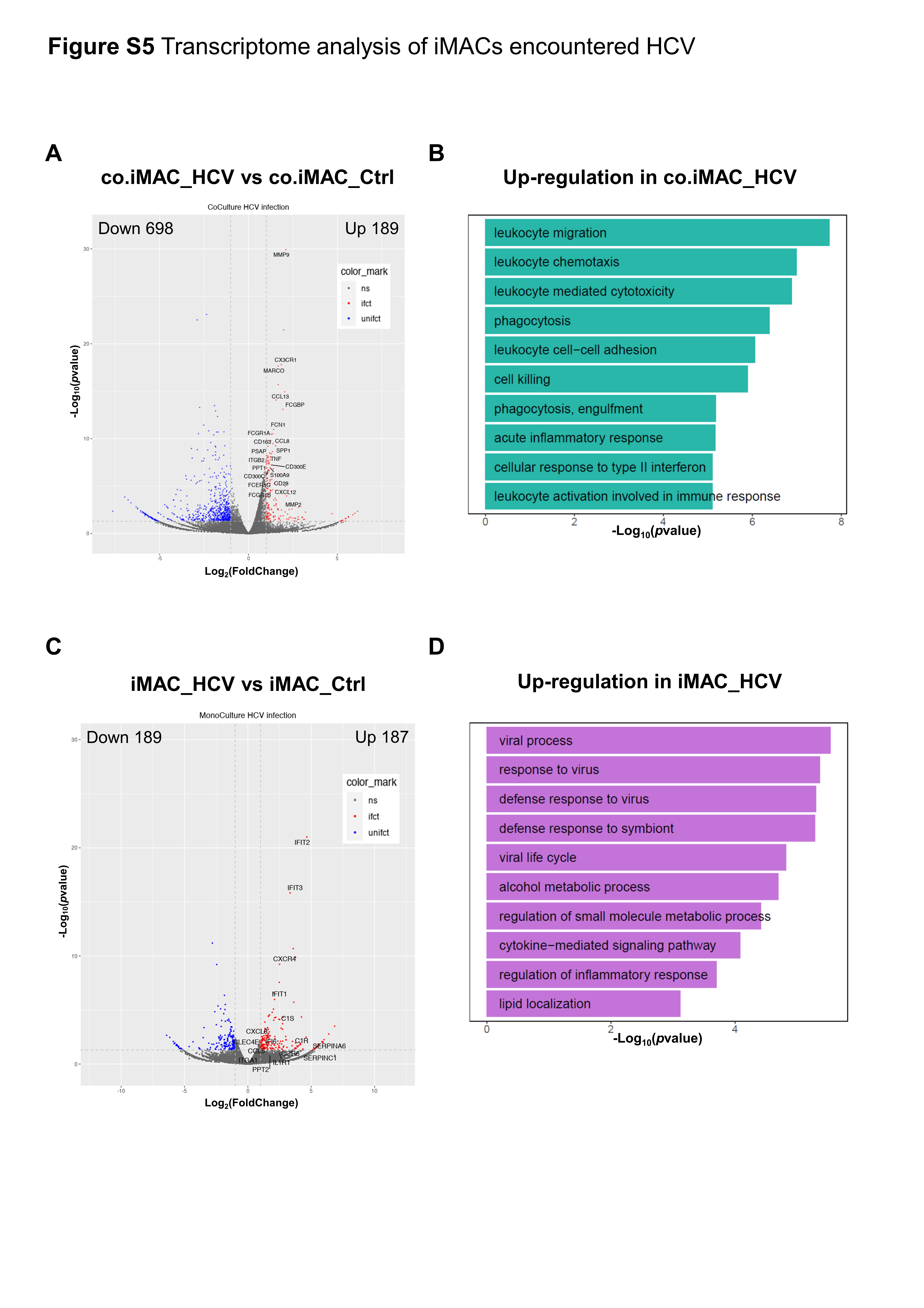

Supplement: lnae001_suppl_Supplementary_Figures_S5 [file lnae001_suppl_Supplementary_Figures_S5.TIF]

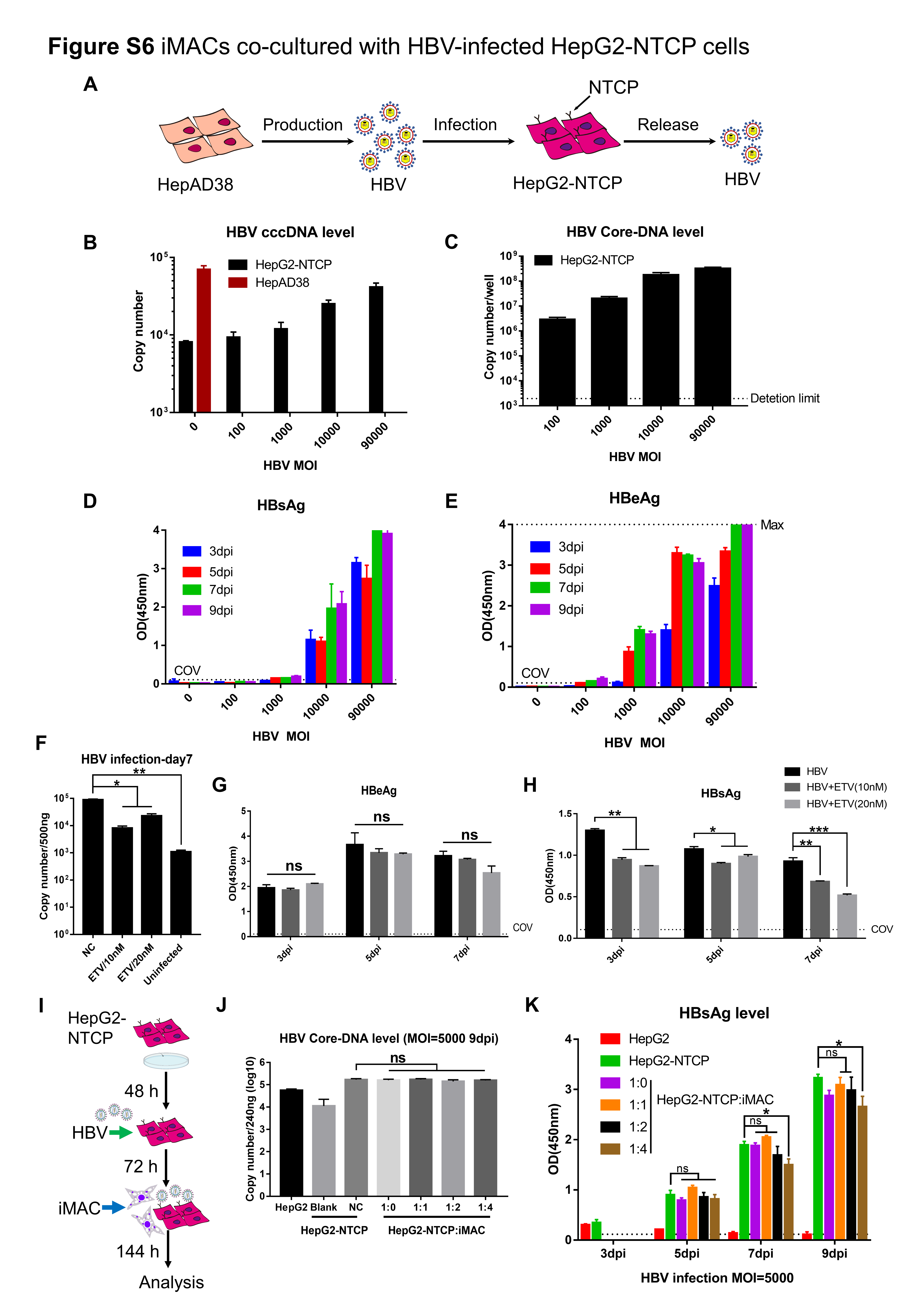

Supplement: lnae001_suppl_Supplementary_Figures_S6 [file lnae001_suppl_Supplementary_Figures_S6.TIF]
