# Supplementary material for: Modeling antiviral response in the liver using human pluripotent stem cell-derived macrophages
Source: Life Med. 2024 Jan 12;3(1):lnae001. doi: 10.1093/lifemedi/lnae001 (PMC11749128; doi:10.1093/lifemedi/lnae001)
Supplement: lnae001_suppl_Supplementary_Material [file lnae001_suppl_Supplementary_Material.docx]

# Modeling antiviral response in the liver using human pluripotent stem cell-derived macrophages

# Supplemental Methods

## Cell culture

H1 cells and induced pluripotent stem cells (iPSCs) were maintained on Growth Factor Reduced Matrigel (Corning)-coated well plates in TeSR-E8 (StemCell Technologies) medium. They were passageed with ESGRO Complete Accutase (Millipore) and seeded onto Matrigel- coated well plates in TeSR-E8 medium supplemented with 10 mM ROCK inhibitor Y27632 (Selleck) on the first day.

Huh7 cells, H2B-RFP transgenic Huh7 cells, Huh7.5.1 cells, HepAD38 cells, and HepG2- NTCP cells were routinely maintained in DMEM medium (Gibco) containing 10% (*v*/*v*) fetal bovine serum (FBS) (Biological Industries), 1% (*v*/*v*) GlutaMAX Supplement and 100 U/mL Pen Strep (Gibco).

Cryopreserved primary human hepatocytes (pHHs) (catalog number: BQH1000.H15+) were purchased from XenoTech (USA). Cryopreserved pHH vials were removed from liquid nitrogen and immediately immersed into the 37°C water bath until the ice almost melted. Then the pHH suspension was transferred into a 15 mL centrifuge tube containing 10 mL OptiThaw hepatocyte medium (XenoTech), and the tube was gently inverted 2–3 times. pHHs were spun down for 5 min at 200 *g* at room temperature. The supernatant was discarded, and pHHs were resuspended with 5 mL OptiPlate hepatocyte medium (Xenotech). The cell suspension was added to a collagen type I coated 24-well plate at a density of 2×105 cells/cm2. pHHs were cultured for 24 h before co-culture. All cells were cultured in a humidified cell incubator at 5% CO2 and 37°C.

## Macrophage differentiation

Macrophage differentiation was done as previously described [[1](#_ENREF_1)] with slight modifications. In brief, undifferentiated H1 cells or iPSCs cultured in TeSR-E8 medium were dissociated into single-cell suspension with Accutase and plated onto a Matrigel-coated 12-well plate at a density of 3×104 cells/cm2 in TeSR-E8 medium supplied with 10 mM Y27632. 24 h after plating, the culture medium was replaced with AATS medium supplemented with 5 ng/mL human BMP4 (PeproTech) for 24 h. Then, 2 μM GSK3 inhibitor CHIR99021 (Selleck) was added to the AATS medium with 5 ng/mL BMP4, and cells were cultured for another 48 h. After that, the whole culture was dissociated with Accutase and replated onto another Matrigel-coated 12-well plate at a density of 7×104 cells/cm2 in RPMI 1640 medium (Gibco) supplied with 2% B27 (*v*/*v*) Supplement (Gibco), 50 mg/mL ascorbic acid (Sigma-Aldrich), 1% (*v*/*v*) GlutaMAX Supplement, 1% (*v*/*v*) MEM Non-Essential Amino Acids (NEAA) Solution, 50 ng/mL human VEGF165 (Sino Biological), and 10 ng/mL bFGF (Sino Biological), and cells were cultured for 48 h. Afterward, 10 μM ALK5 inhibitor SB431542 (Selleck) was added for another 72 h. The floating hematopoietic stem/progenitor cells (HSPCs) were collected and transferred to another 6-well plate at a density of 1×105 cells/cm2 in RPMI 1640 medium supplied with 2% (*v*/*v*) B27 Supplement, 50 mg/mL ascorbic acid, 1% (*v*/*v*) GlutaMAX Supplement, 1% (*v*/*v*) MEM NEAA Solution, 50 ng/mL recombinant human M-CSF (Novoprotein) and 10 ng/mL recombinant human IL-3 (Novoprotein), and cells were cultured for 7 days. From day 7 onwards, the medium was changed to RPMI 1640 with 10% (*v*/*v*) non-heat-inactivated FBS, 1% (*v*/*v*) GlutaMAX Supplement, 1% (*v*/*v*) MEM NEAA Solution, and 25 ng/mL recombinant human M-CSF (Novoprotein) and cultured for another 3 days, after which the floating cells were washed away, and the adherent cells were exclusively iMACs.

## Flow cytometry analysis

Cells or cultures were dissociated into single-cell suspension with Accutase and filtered through a 70 μm cell strainer (BD Biosciences). Single cells were spun down at 4°C 300 *g* for 3 min and resuspended with 100 μL FACS buffer composed of PBS and 2% (*v*/*v*) FBS. The desired antibodies were added to the cell suspension (usually 1:100 diluted) and incubated at 4°C for 30 min away from light. Then cells were washed with 1 mL FACS buffer 3 times and resuspended in 300 μL FACS buffer. 1 ng/μL DAPI was added into the final cell suspension to distinguish dead cells, and cells were directly loaded onto the flow cytometer. The flow cytometry data were collected on a BD FACSAria SORP cytometer (BD Biosciences) and analyzed using FlowJo software (v10.4) (TreeStar).

## Single-cell RNA-seq and single-cell ATAC-seq library preparation

Differentiated cells on day 14 were dissociated with Accutase and filtered through a 70 μm cell strainer to obtain a single-cell suspension. Cells were spun down at 4°C 300 g for 3 min and resuspended with FACS buffer to a final concentration of 2×10^6^ cells/mL. Live/dead assay showed around 85% of the cells were viable. scRNA-seq and scATAC-seq library preparation and sequencing were done by CapitalBio Technology [(https://www.capitalbiotech.com/)](http://www.capitalbiotech.com/)). For scRNA-seq, the cell suspension was immediately loaded onto the Chromium Single Cell Controller (10× Genomics) for droplet formation. cDNA and subsequent sequencing library were generated using Chromiun Single Cell 3’ Reagent Kit (v3 Chemistry) (10× Genomics) according to the instruction manual. For sc-ATAC-seq, cell nuclei were extracted with Chromiun Nuclei Isolation Kit (v2 Chemistry) (10× Genomics) and then loaded onto the Chromium Single Cell Controller for droplet formation. Sequencing libraries were generated using Chromium Single Cell ATAC Reagent Kit (v2 Chemistry) (10× Genomics) according to the manufacturer’s protocol. The scRNA-seq and scATAC-seq libraries were sequenced using HiSeq X Ten sequencer (Illumina) separately with a paired-end 150 bp (PE150) strategy.

## Single-cell RNA-seq and single-cell ATAC-seq data processing and analysis

The scRNA-seq and scATAC-seq data of iMAC differentiation are accessible through GEO accession number GSE234577. The quality of raw sequencing data was checked using FastQC (v0.11.9). Single-cell gene expression and peak accessibility matrices were generated following cellranger count (v3.1.0) and cellranger-ATAC (v1.2.0) pipelines. These resulting unique molecular identifier (UMI) matrices were used in the following cell clustering analysis. For integrative analysis of multiple samples, the cellrange aggr (v2.0.1) pipeline was used to normalize samples to equal read depth.

For scRNA-seq cell clustering and identification of differentially expressed genes, cell type identification was done using the R package Seurat (v4.0). In brief, cells were filtered using 3 criteria: number of expressed genes, number of UMI counts, and percentage of mitochondrial genes in each cell. Cells with any of these metrics falling 2 standard deviations of all cells were discarded. Next, highly variable genes were identified and used for principal component analysis. 50 principal components were used to perform cell clustering using FindClusters function in Seurat with the resolution parameter set to 0.8. Differentially expressed genes were identified using FindAllMarkers function, comparing each cell cluster against the rest of all cells.

For scATAC-seq cell clustering and identification of differentially accessible peaks, cell type identification was performed using the R package Signac (v1.1.0). Briefly, cells with less than 2000 ATAC-seq fragments or less than 1000 peaks were filtered out. We next utilized the latest semantic indexing procedure in Signac package to normalize and reduce the dimension of scATAC-seq data. 2 to 30 latent semantic indexing (LSI) components were used to perform cell clustering using FindClusters function in Seurat with the resolution parameter set to 0.8. Cell annotations were transferred from the scRNA-seq dataset by adopting FindTransferAnchors and TransferData functions provided in the Seurat package.

Cell cycle analysis was performed using the CellCycleScoring function in the Seurat package with a previously curated set of G1/S and G2/M genes [[2](#_ENREF_2)]. We evaluated the cell cycle status of each cell according to the average expression of these two cell cycle gene sets. We tested various thresholds to identify proliferative cells and found that the difference in cell cycle activities between cell clusters is robust against threshold change. Cell fate trajectory analysis was performed using RNA Velocity according to the instructions [[3](#_ENREF_3)].

## Huh7 and iMAC co-culture assay

For Huh7-iMAC co-culture, Huh7 cells were first seeded in a 24-well plate at a density of 2×104 cells/cm2 in DMEM medium containing 10% (*v*/*v*) FBS, 1% (*v*/*v*) GlutaMAX Supplement and 100 U/mL Pen Strep. 24 h later, the culture medium was changed to a co-culture medium composed of DMEM with 10% (*v*/*v*) non-heat inactivated FBS, 1% (*v*/*v*) GlutaMAX Supplement, 1% (*v*/*v*) MEM NEAA Solution, and 25 ng/mL recombinant human M-CSF. iMACs were added to the co-culture medium at 2:1, 1:1, or 1:2 ratios (iMACs to Huh7 cells). After 24 h, the co-culture medium with unattached iMACs was removed, and fresh co-culture medium was added. Cells were cultured for another 48 h. After that, the whole culture was dissociated with Accutase. The RFP–CD163+ iMACs and RFP+ Huh7 cells in the culture were sorted by fluorescence-activated cell sorting (FACS) for RNA extraction and RNA-seq analysis.

## pHH and iMAC co-culture assay

For pHH-iMAC co-culture, pHHs were first seeded in a 24-well plate at a density of 2×105 cells/cm2 in the OptiPlate hepatocyte medium. 24 h later, the culture medium was changed to a co-culture medium containing 50% of OptiPlate hepatocyte medium and 50% of iMAC differentiation medium (RPMI1640 with 2% (*v*/*v*) B27 Supplement, 50 mg/mL ascorbic acid, 1% (*v*/*v*) GlutaMAX Supplement, 1% (*v*/*v*) MEM NEAA Solution, 50 ng/mL recombinant human M-CSF and 10 ng/mL recombinant human IL-3). Then sorted CD14+CD163+ iMACs were seeded at a 1:2 ratio (iMACs to pHHs) into the co-culture medium. Half of the co-culture medium was changed every day. After 3 or 5 days of co-culture, the culture was dissociated with Accutase. The CD163+ iMACs and CD163– pHHs in the culture were sorted by FACS for RNA extraction and RNA-seq analysis.

## Immunofluorescence

Cells in culture wells or plates were washed with PBS 3 times and were fixed *in situ* with 4% paraformaldehyde at room temperature for 10 min. Then they were permeabilized in a permeabilization buffer, which contained 0.2% Triton X-100 (Sigma) in PBS, for 10 min, and blocked in block buffer (permeabilization buffer with 5% (*w*/*v*) bovine serum albumin (BSA) (Sigma-Aldrich) added) for another 30 min. Cells were then incubated in primary antibodies at 4°C overnight and detected by DyLight 488- or 549-conjugated secondary antibodies (Thermo). Nuclei were stained with 1 μg/mL DAPI (Sigma-Aldrich). All antibodies and DAPI were diluted with the block buffer and washed 3 times after antibody incubation. A Nikon TiE fluorescence microscope was used for image acquisition.

## RNA extraction and next-generation RNA-seq library preparation

Cells were sorted based on surface antigen, spun at 4°C for 3 min, and lysed by TRIzol (Ambion). RNA extraction was done according to the instruction manual. For high-throughput RNA-seq, RNA libraries were constructed according to the Smart-seq2 protocol [[4](#_ENREF_4)]. The RNA libraries were sequenced by a NovaSeq 6000 sequencer (Illumina) with PE150 strategy at Novogene (cn.novogene.com).

## Bulk RNA-seq data analysis

The bulk RNA-seq datasets of iMACs co-culture are accessible through GEO accession number GSE234572. Reads were trimmed with the Trim Galore (v0.6.7) software with the default parameters. Then adaptor-trimmed paired-end RNA-seq reads were mapped to the GRCh38 human genome using hisat2 (v2.2.1) with default parameters. The read counts were calculated using featurecount (v2.0.3) with extra parameters -O -t exon apart from the default parameters. The raw read count normalization and differentially expressed gene (DEG) calculation were obtained using DESeq2 (v1.38.3 software) with the following criteria (|log2FC| > 1 and *p*adj < 0.05). Kupffer cell-enriched gene list was obtained from a recently published single-cell RNA-seq study of human liver and intrahepatic macrophages [[5](#_ENREF_5), [6](#_ENREF_6)].

## Enrichment analysis

DEG lists for enrichment analyses were obtained using DESeq2 as mentioned above, while the criteria were changed to (|log2FC| > 0.2 and *p*adj < 0.05) to obtain more gene ontology (GO) or gene set enrichment analysis (GSEA) terms. The R package clusterProfiler (v4.6.2) was used to perform gene ontology enrichment with default arguments. GO terms for each cluster were re-summarized and visualized as bar plots using a customized script, and FDR- adjusted *p*-value, *q*-value were plotted to show the significance. GSEA was performed with the GSEA function embedded in clusterProfiler using the gene sets from Human MsigDB collections (Canonical pathways derived from KEGG pathways, www.gsea- msigdb.org/gsea/msigdb).

## HCV production, concentration, and titration

Methods for hepatitis C virus (HCV) production, titration, and concentration have been previously described [[7](#_ENREF_7)]. In brief, a GFP coding sequence was inserted into the pJFH-1 plasmid containing HCV cDNA, and the GFP-HCV genomic RNA was transcribed *in vitro* from the linearized plasmid. Huh7.5.1 cells were then transfected with the GFP-HCV genomic RNA. The transfected Huh7.5.1 cells were further cultured and passaged before 100% confluency. The culture supernatants from the second and third week post- transfection were collected. The culture supernatants containing HCV were centrifuged and filtered to remove cellular debris, then the viruses were concentrated by PEG8000, aliquoted, and stored at –80°C.

Virus titration was performed by inoculating Huh7.5.1 cells with 10-fold serially diluted virus samples. After a 3-day infection, the level of virus infection was examined based on HCV NS5A immunofluorescence. The viral titer is measured as focus-forming units per milliliter of supernatant (ffu/mL), determined by the average number of NS5A-positive foci detected at the highest dilutions.

## HCV infection of Huh7-iMAC co-culture

For the Huh7-iMAC co-culture infection assay, iMACs were added to Huh7 cells before or after GFP-HCV infection, as shown in schematics. GFP-HCV was added in the co-culture at a multiplicity of infection (MOI) = 0.5. The inoculum was changed, and the culture was washed twice with DMEM medium 12 hours post-infection (hpi) before adding the fresh co- culture medium. Then cells were grown for another 60 h. The supernatants of the co-culture were collected every day. After that, cells were analyzed by immunofluorescence staining or flow cytometry (FCM) analysis. As for the co-culture post-infection, Huh7 cells were first infected by GFP-HCV for 12 h, then cultured in fresh medium without virus for 36 h. After a 2-day infection, cells were dissociated and seeded into new 24-well plates, and iMACs were added. Supernatants of the co-culture or mono-culture were collected every day. After 96 h of co-culture, cells were analyzed by immunofluorescence staining or FCM analysis.

## HBV production and infection of HepG2-NTCP and iMACs co-culture

HepAD38 cell line with a tetracycline-inducible hepatitis B virus (HBV) genome inserted into the genome was used to produce HBV according to a previously reported method [[8](#_ENREF_8)]. HepG2 cells constitutively expressing Na+ taurocholate cotransporting polypeptide (NTCP) were constructed. HBV was added to the HepG2-NTCP cells at various concentrations. The copy number of HBV covalent closed circular DNA (cccDNA) was determined by Q-PCR. HBV-specific surface antigen (HBsAg) and envelop antigen (HBeAg) were quantified by enzyme-linked immunosorbent assay (ELISA). Entecavir (ETV), an inhibitor of HBV infection, was used as a positive control. iMacs were added to HepG2-NTCP cells 72 h after HBV infection and cultured for 96 h before cccDNA and HBsAg quantification.

All steps in this part were performed in a BSL-2 laboratory in Qiang Ding’s lab.

## Biosafety declaration

All experiments and operations involving HCV or HBV were approved to be performed in a biosafety level (BSL)-2 laboratory in Qiang Ding’s lab, which obtained registration from the Municipal Health Commission of Haidian District in Beijing in 2021.

## Statistical analysis

All column or scatter plots were generated using Prism 7 and all statistical analyses were performed in Prism 7. The columns or scatters in plots represented mean values of the results, and the bars indicated the standard deviation (S.D.) of each group. Independent *t*-test was used for comparison in tests with only two variables, such as those in Fig. 2D–F. One-way ANOVA and multiple *t*-test were used for comparison in tests with more than two variables, such as those in Fig. 2, Fig. S4, and Fig. S6.

## Data availability

The scRNA-seq and scATAC-seq data of iMAC differentiation and the bulk RNA-seq datasets of iMACs co-culture are accessible through GEO accession number GSE234577 (Go to www.ncbi.nlm.nih.gov/geo/que[ry/acc.cgi?acc=GSE234577,](http://www.ncbi.nlm.nih.gov/geo/query/acc.cgi?acc=GSE234577) enter token utezieaehtsdrov into the box) and GSE234572 (Go to www.ncbi.nlm.nih.gov/geo/que[ry/acc.cgi?acc=GSE234572,](http://www.ncbi.nlm.nih.gov/geo/query/acc.cgi?acc=GSE234572) enter token mxmrkegyhzsbvkv into the box). Data supporting this study’s findings have been included within the paper and the Supplementary information.

## References

1. Duan, F., et al., *Biphasic modulation of insulin signaling enables highly efficient hematopoietic differentiation from human pluripotent stem cells.* Stem Cell Res Ther, 2018. **9**(1): p. 205.

2. Tirosh, I., et al., *Dissecting the multicellular ecosystem of metastatic melanoma by single-cell RNA-seq.* Science, 2016. **352**(6282): p. 189-96.

3. La Manno, G., et al., *RNA velocity of single cells.* Nature, 2018. **560**(7719): p. 494-498.

4. Picelli, S., et al., *Full-length RNA-seq from single cells using Smart-seq2.* Nat Protoc, 2014. **9**(1): p. 171-81.

5. Aizarani, N., et al., *A human liver cell atlas reveals heterogeneity and epithelial progenitors.* Nature, 2019. **572**(7768): p. 199-204.

6. MacParland, S.A., et al., *Single cell RNA sequencing of human liver reveals distinct intrahepatic macrophage populations.* Nat Commun, 2018. **9**(1): p. 4383.

7. Lindenbach, B.D., et al., *Complete replication of hepatitis C virus in cell culture.* Science, 2005. **309**(5734): p. 623-6.

8. Xu, R., et al., *Advances in HBV infection and replication systems in vitro.* Virol J, 2021. **18**(1): p. 105.

**Supplemental Figure Legends**

## Supplemental Figure 1. Marker gene expressions and cell cycle profiles of different cell clusters.

(A) UMAP plots of selective marker gene expressions in different cell clusters. (B) Cell-cycle profile analysis of different cell clusters.

## Supplemental Figure 2. Cells clustered by transcriptomes in iMAC-hepatic cell co- cultures.

(A) Heatmap showing Euclidean distance among each sample. The transcriptome of sorted iMACs, pHHs, and Huh7 cells from the co-culture assay separated clearly. (B) Heatmaps with the left panel showing hepatocyte and macrophage marker gene expressions of different cell types in iMAC-pHH co-cultures and the right panel showing those in iMAC-Huh7 cell co-cultures. Macrophage marker genes, *CD14*, *CD163*, *CSF1R*, etc., were highly expressed in iMACs in left and right panels. Hepatocyte-specific genes, *ALB*, *KRT18*, *CYP3A5*, etc., were specifically expressed in pHHs or Huh7 cells in left or right panel, respectively.

## Supplemental Figure 3. Transcriptome analysis of iMACs co-cultured with hepatic cells.

(A) GO analysis showing significantly up-and down-regulated genes in iMACs after co-culture with Huh7 cells. Up-regulated genes are involved in the regulation of response to wounding, and liver development genes are enriched. Down-regulated genes are related to leukocyte migration, antigen processing, vacuole organization, and metabolic processes. (B) GO analysis of significantly up- and down-regulated genes in iMACs after co-culture with pHHs. Similar GOs were found in (A). (C) GSEA on complement and coagulation cascades, and metabolism pathways related to cytochrome P450 in iMACs after co-cultured with Huh7 cells. (D) GSEA on metabolism pathways related to organic acids in iMACs after co-cultured with pHH. (E) Venn plots of up- and down-regulated genes in iMACs after co-culture with Huh7 cells and pHHs. 1725 and 953 genes in iMACs were up- and down-regulated after co-culture with Huh7 cells. While 2081 and 1087 genes in iMACs were up- and down-regulated after co-culture with pHHs. (F) GO analysis of 643 and 303 shared up- and down-regulated genes in iMACs after co- culture with Huh7 cells and pHHs. Up-regulated genes are mostly involved in wound healing, metabolic processes, hepatic system development, cholesterol homeostasis, etc. Down- regulated genes regulate antigen processing, reactive oxygen species metabolic processes, membrane lipid processes, etc. (G) Heatmap of marker genes in iMACs after co-culture with Huh7 cells and pHHs. Kupffer cell-enriched gene list was obtained from a recently published single-cell RNA-seq study of human liver and intrahepatic macrophages [[5](#_ENREF_5), [6](#_ENREF_6)]. In (A), (B), and (F), “Up” or “Down” indicates the GO terms enriched by the up-regulated or down-regulated genes in co-cultured iMACs compared with non-cultured iMACs, respectively. *N* = 2 biological replicates were performed in the transcriptome profiling and analysis.

## Supplemental Figure 4. iMAC inhibition of HCV infection depends on cell-cell contact.

(A) Schematic view of transwell co-culture design and representative results of FCM analysis. (B) Bar graph quantification of HCV-infected cells in different transwell co-culture groups indicated in (A). *N* = 3 biological replicates. The bar plot in (B) shows the mean values of the results, and the bars represent the S.D. of each group. One-way ANOVA and multiple t-test were used for significance analysis in (B). ** represents *p* < 0.01, and “ns” denotes “no significance.” All statistical analyses were performed by Prism 7.

## Supplemental Figure 5. Transcriptome analysis of iMACs encountered HCV.

(A) Volcano plot showing significantly up- and down-regulated genes in iMACs co-cultured with HCV-infected versus non-infected Huh7 cells. (B) Bar plot showing GO terms enriched by up-regulated genes in iMACs co-cultured with HCV-infected Huh7 cells. (C) Volcano plot showing significantly up- and down-regulated genes in iMACs encountered HCV versus iMACs alone. (D) Bar plot showing GO terms enriched by up-regulated genes in iMACs encountered HCV. “ns” indicated non-significant genes, “ifct” indicated HCV-infected or added, and “unifct” indicated no HCV infected or added.

## Supplemental Figure 6. iMACs co-cultured with HBV-infected HepG2-NTCP cells.

(A) Schematics of HBV production and infection. (B) HBV cccDNA level in the infected HepG2-NTCP cells with different HBV multiplicity of infection (MOI). Data of HepAD38, the cell line for HBV production, at 0 MOI was added as a comparison. (C) HBV core DNA level in the infected HepG2-NTCP cells with different HBV MOIs. (D) HVB surface antigen (HBsAg) levels in the infected HepG2-NTCP cells with different HBV MOIs at various time points post-infection. (E) HBV envelop antigen (HBeAg) levels in the infected HepG2-NTCP cells with different HBV MOIs at indicated time points post-infection. (F) HBV core DNA levels in the HBV-infected HepG2-NTCP cells under different concentrations of entecavir (ETV) treatments. (G) HBeAg levels in the HBV-infected HepG2-NTCP cells after ETV treatments. (H) HBsAg levels in the HBV-infected HepG2-NTCP cells after ETV treatments. (I) Schematic illustration of HBV infection of iMAC-HepG2-NTCP co-culture. (J) HBV core DNA levels in the cell lysates from the whole infected co-cultures. (K) HBsAg levels in the infected iMAC-HepG2-NTCP co-cultures with different HepG2-NTCP to iMAC ratios at different time points post-infection. *N* = 3 biological replicates for tests in (B–H) and (J–K). The bar plots show the mean values of the results, and the bars represent the S.D. of each group. * represents *p* < 0.05, ** represents *p* < 0.01, *** represents *p* < 0.001, and “ns” represents “no significance.” One-way ANOVA and multiple *t*-test were used for significance analysis in (F–H and J). All statistical analyses were performed by Prism 7. “COV” represented the coefficient of variation. “dpi” represented the day post-infection.
